# Effects of riboflavin/ultraviolet-A scleral collagen cross-linking on regional scleral thickness and expression of MMP-2 and MT1-MMP in myopic guinea pigs

**DOI:** 10.1371/journal.pone.0279111

**Published:** 2023-01-18

**Authors:** Xiaotong Lv, Lingbo Lai, Yushan Xu, Mingshen Sun, Yu Li, Yanzheng Song, Ningli Wang, Fengju Zhang

**Affiliations:** Beijing Tongren Eye Center, Beijing Tongren Hospital, Capital Medical University, Beijing, China; University of Hong Kong, HONG KONG

## Abstract

**Objective:**

To investigate the effects of scleral collagen cross-linking (SXL) using riboflavin and ultraviolet A (UVA) light on the scleral thickness of different regions and expression of matrix metalloproteinase 2 (MMP-2) and membrane-type MMP-1 (MT1-MMP) in guinea pigs with lens-induced myopia.

**Methods:**

Forty-eight 4-week-old guinea pigs were assigned to three groups (n = 16 per group): SXL group, lens-induced myopia (LIM) group, and control group. The sclera of the right eye of the guinea pig in the SXL group was surgically exposed, riboflavin was dropped on the treatment area for 10 minutes before the 30-minute UVA irradiation. The same surgical procedure was performed in the LIM group without UVA irradiation. The -10.00 D lenses were then placed on the right eyes of guinea pigs in the SXL and LIM groups for six weeks. The control group received no treatment. The left eyes were untreated in all groups. The ocular axial length (AXL) and refraction were measured at 4 weeks and 10 weeks of age. 10-week-old guinea pigs were sacrificed, and the right eyes were enucleated and evenly divided for preparation of hematoxylin and eosin (HE) stained sections, quantitative real-time polymerase chain reaction (qPCR) and western blotting. The scleral thickness of different regions was measured on HE stained sections. The temporal half of the sclera was harvested to measure the expression of MMP-2 and MT1-MMP by qPCR and western blotting.

**Results:**

The AXL was significantly shorter, and the degree of myopic refraction was significantly lower in the SXL group than those in the LIM group at 10 weeks of age. The scleral thickness of the cross-linked area was significantly greater in the SXL group than that of the corresponding area in the LIM group, while the scleral thickness of the untreated nasal side was not significantly different between the SXL group and the LIM group. The expression of MMP-2 and MT1-MMP of the cross-linked sclera was significantly downregulated compared with that of the corresponding area in the LIM group.

**Conclusion:**

Riboflavin/UVA SXL could slow myopia progression and thicken the cross-linked sclera in guinea pigs, which might be related to the downregulation of MMP-2 and MT1-MMP expression during the scleral remodeling process.

## Introduction

Myopia is a major public health concern worldwide with the increasing prevalence. The global prevalence of myopia is predicted to increase to 49.8% by 2050, and the prevalence of high myopia is expected to be 9.8% [1]. Scientists have realized the crucial role of scleral remodeling in eye size and myopia development [2]. Excessive ocular axial length (AXL) elongation and scleral thinning, as important features of progressive myopia, are considered to result from an imbalance between scleral extracellular matrix (ECM) synthesis and degradation during scleral remodeling [2–4]. Matrix metalloproteinases (MMPs) play a crucial role in the degradation of collagen and other ECM components [5]. Matrix metalloproteinases-2 (MMP-2) is secreted as a latent proenzyme named proMMP-2, which needs to be activated to degrade ECM components. Activation of proMMP-2 involves the formation of a trimolecular complex with membrane-type matrix metalloproteinase-1 (MT1-MMP) and TIMP-2 on the cell surface [6]. Increased MMP-2 expression and activity are observed in both mammalian [7, 8] and avian [9, 10] models of myopia. An increase in MMP-2 expression has also been found in the aqueous humor [11] and vitreous [12] specimens from human patients with high myopia.

Since myopia is the leading cause of visual impairment, effective and safe therapeutic interventions to prevent myopia-related complications and vision loss are urgently needed. Studies on human patients with high myopia and experimental myopia models have shown that the sclera is biomechanically weakened during the development of myopia [13–15]. Collagen cross-linking has been used in multiple settings to mechanically stabilize collagenous tissues [16, 17]. Scleral collagen cross-linking (SXL) has therefore been proposed as a potential treatment for myopia [18]. Wollensak and colleagues [19] reported that the scleral stiffness increased by scleral collagen cross-linking using riboflavin and ultraviolet A (UVA) light remained effective for up to 8 months in rabbits. In this process, UVA (370 nm) activates riboflavin by converting it to a triplet state, and activated riboflavin in turn produces reactive oxygen species (ROS), which react with collagen fibril molecules. New chemical bonds are formed between the amino of the collagen fibril molecules and enhance the biomechanical properties of the sclera [20]. Riboflavin/UVA SXL has been proven to slow AXL elongation and decrease myopic refractive error in a rabbit model of form deprivation-induced myopia and a guinea pig model of lens-induced myopia [21, 22].

However, the mechanism by which riboflavin/UVA SXL influences the scleral remodeling process to control progressive myopia has not yet been elucidated. In this study, we performed riboflavin/UVA SXL in guinea pigs with lens-induced myopia. Regional scleral thickness was measured and the expression of MMP-2 and MT1-MMP in the sclera was assessed to explore the effect of riboflavin/UVA SXL on scleral remodeling.

## Methods

### Animals

Male pigmented guinea pigs (Cavia porcellus) aged 4 weeks and weighing between 200–250 g were used in this study. They were then housed in groups of 3–4, in opaque plastic boxes (65 × 45 × 20 cm) with wire mesh lids and a floor covered with wood shavings. Food and water (supplemented with vitamin C) were available ad libitum. The ceiling lights were on a 12 h light/12 h dark cycle. The room temperature was 24–26 ℃. The treatment and care of the animals were conducted in accordance with the Association for Research in Vision and Ophthalmology (ARVO) Statement for the Use of Animal in Ophthalmic and Vision Research. All experimental procedures were reviewed and approved by the Institutional Animal Care and Use Committee of Nankai Hospital (NKYY-DWLL-2019-109).

### Experimental procedure

**Fig 1:**
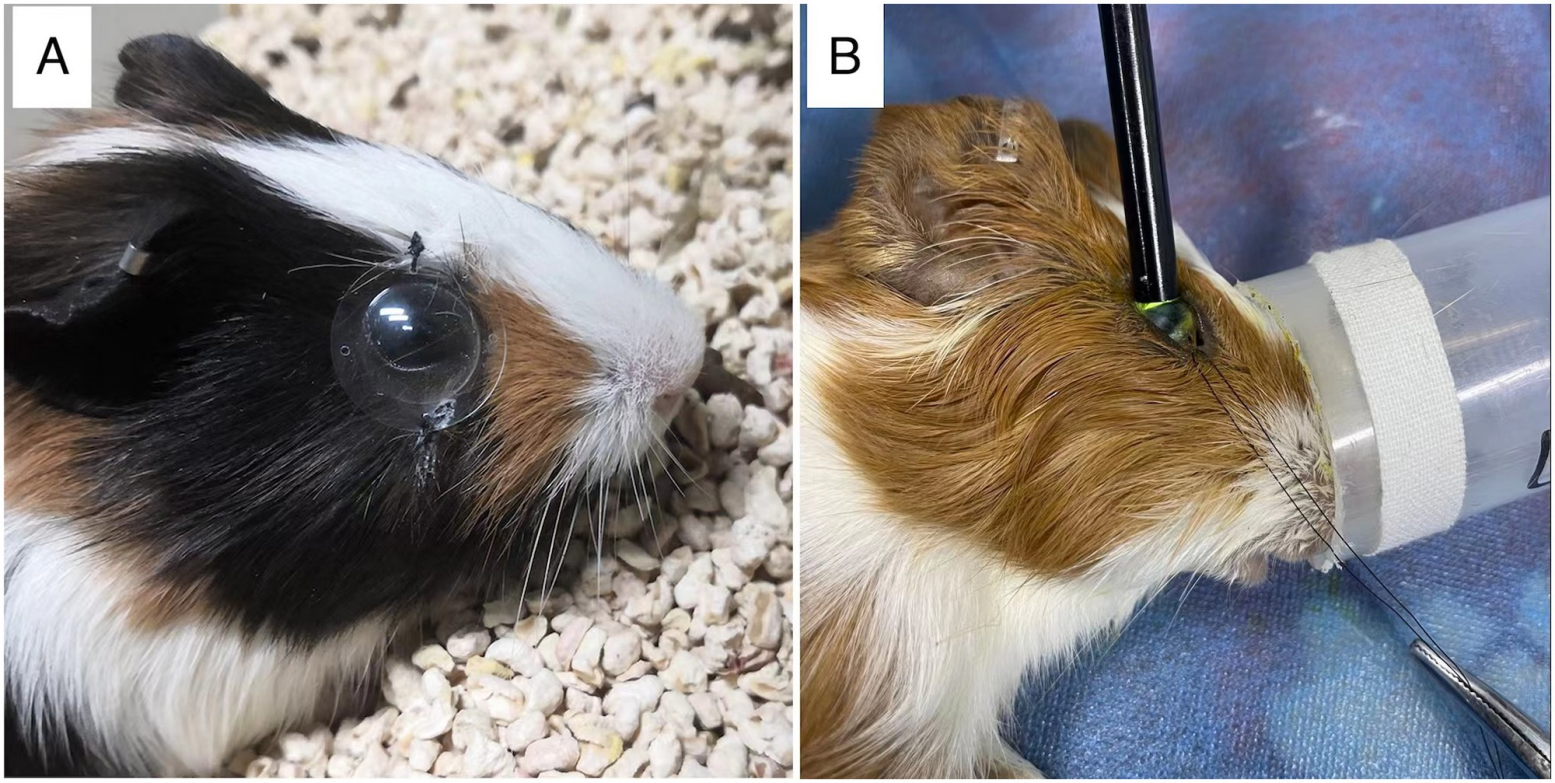
Lens induction and scleral collagen cross-linking procedure. (A) A -10.00 D lens was sutured and fixed to the right eye of each guinea pig. (B) A circular UVA irradiation probe (4 mm diameter) was placed 3.5 mm posterior to the temporal corneal limbus of the right eye of each guinea pig.

A total of forty-eight 4-week-old guinea pigs were randomly divided into three groups (n = 16 per group). In all groups, the left eye was untreated. Neither eye underwent manipulation in the control group. SXL using 0.1% riboflavin and UVA (3.0 mW/cm^2^, 30 minutes) was performed on the right eye of guinea pigs in the SXL group, following the surgical procedure described in the next section. The guinea pigs in the LIM group were subjected to the same procedure without UVA irradiation. After the surgery, -10.00 D lenses made of polymethylmethacrylate (diameter, 12 mm; optic zone, 10.5–11.5 mm) were placed in front of the right eyes of the guinea pigs in the LIM and SXL groups as shown in Fig 1A. The distance from the cornea to the lens apex was approximately 5 mm. The lens was worn continuously for six weeks except when it was removed for cleaning once a day. At 10 weeks of age, all animal subjects were euthanized by intraperitoneal injection of an overdose of pentobarbital sodium, and their right eyes were enucleated. Five eyeballs from each group were used for hematoxylin and eosin (HE) stained sections, five eyeballs from each group were used for quantitative real-time polymerase chain reaction (qPCR) and six eyeballs from each group were used for western blot analysis.

### Riboflavin/UVA scleral collagen cross-linking

Guinea pigs were anesthetized with isoflurane (induction: 3%, maintenance: 2–2.5%, oxygen flow rate: 1 L/min) during riboflavin/UVA SXL. A suture was placed at the 9 o’clock position of the limbus to move the eyeball. Temporal 180-degree conjunctival peritomy was performed and the temporal equatorial-posterior sclera was exposed as the cross-linking area. A drop of photosensitizer solution containing 0.1% riboflavin (0.1% riboflavin, 20% dextran 500, Peschke D, PESCHKE Trade, Huenenberg, Switzerland) was instilled onto the cross-linking area every minute for 10 minutes before UVA irradiation and then during the 30-minute irradiation period. UVA irradiation (365 nm) with an intensity of 3.0 mW/cm2 (measured by a laser power meter; Lasermate-Q; Coherent, Dieburg, Germany) was applied using a UVA device (UV-X 1000; Avedro Inc., Waltham, MA). A circular UVA irradiation probe (4 mm diameter) was positioned perpendicular to the scleral surface 3.5 mm posterior to the temporal corneal limbus (Fig 1B). The UVA irradiation time was 30 minutes (5.4 J/cm^2^). After the surgery, the conjunctiva was closed with a suture thread (Vicryl 8–0; Ethicon Inc, Scotland, UK). The animals were monitored until they awoke. Tobramycin dexamethasone eye gel (TobraDex, Alcon Inc, Belgium) was applied once per night for three days after the surgery.

### Axial length and refraction measurement

Axial length (AXL) and refraction were measured at baseline (4 weeks of age) and after six weeks of treatment (10 weeks of age). Measurements were taken at the same time of day. Refraction was measured in awake animals in a seated posture. After treatment with 1% cyclopentolate hydrochloride eye drops (Cyclogyl, Alcon, Belgium), spherical equivalent refraction was measured under YZ24 streak retinoscope (6 6 Vision Tech Co., Ltd, Soochow, China). AXL was measured in anesthetized guinea pigs (1.5–2% isoflurane in oxygen) using A-scan ultrasonography (20 MHz). The device was programmed to average the values of 8 acceptable measurements and produce an average reading each time, 5 readings were recorded for each eye and averaged.

### Light microscopy and regional scleral thickness measurement

Before enucleation of the right eyes, a black silk thread was placed in front of the cross-linked area in the SXL group as a marker. Similar black silk threads were placed in front of the corresponding areas in the LIM and control groups. Intact eyeballs were fixed with 4% paraformaldehyde and embedded in paraffin. 4-μm sections were cut horizontally through the marked area. The sections were stained with hematoxylin and eosin and digitized using the Pannoramic Digital Slide Scanner (3DHistech Ltd., Budapest, Hungary).

**Fig 2:**
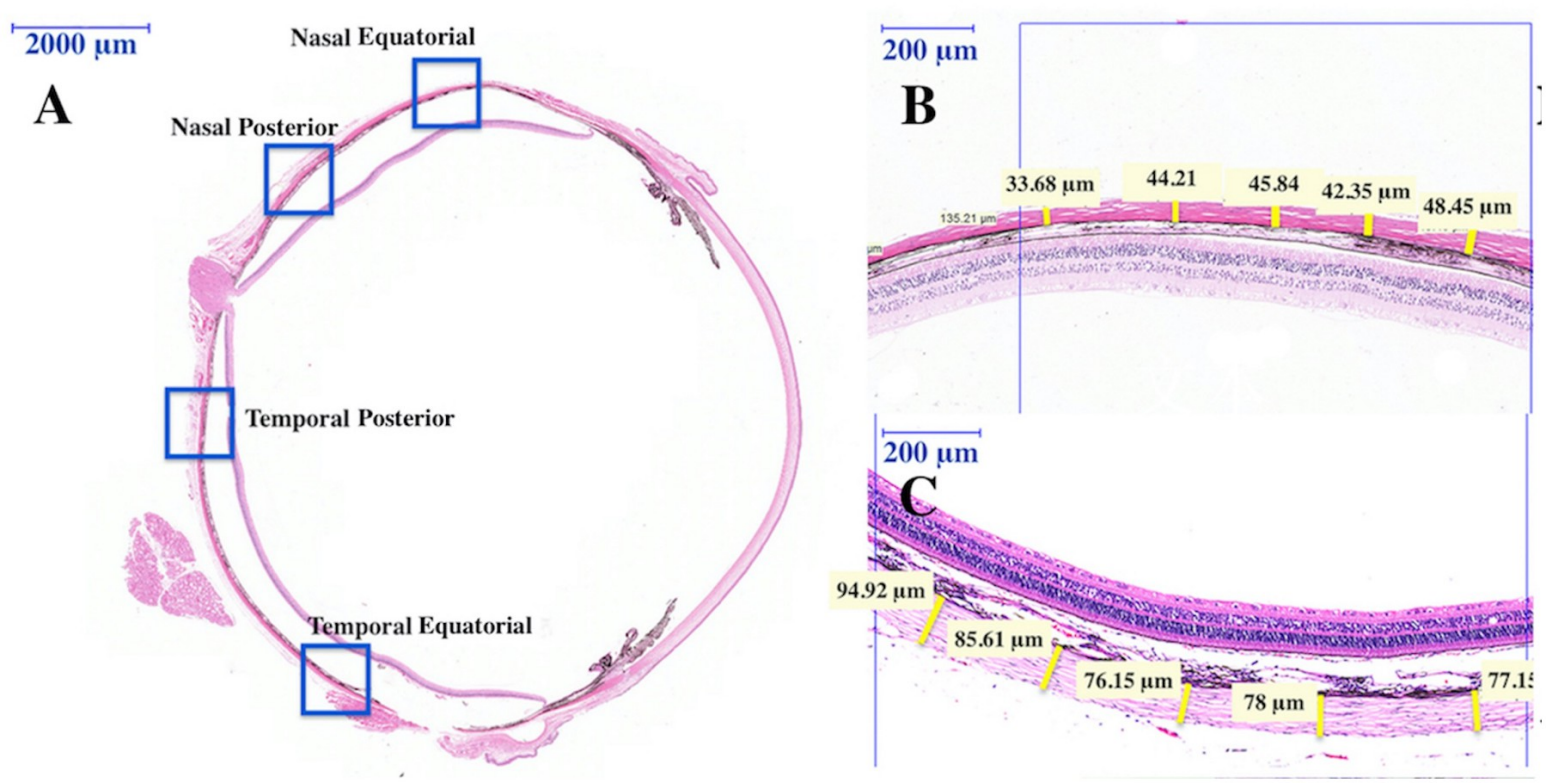
Representative image of a HE stained horizontal section. (A) The regions where the scleral thickness was measured are marked with boxes. Scale bar: 2000 μm. (B) Example of equatorial sclera thickness measurement. Scale bar: 200 μm. **C:** Example of posterior sclera thickness measurement. Scale bar: 200 μm.

The scleral thickness was measured on the HE-stained section using Pannoramic Viewer software (3DHistech Ltd., Budapegroupsst, Hungary). We divided the scans of HE-stained sections into four equal-length segments (1000 μm), which are denoted as the temporal equatorial region, the nasal equatorial region, the temporal posterior region, and the nasal posterior region, as shown in Fig 2A. The equatorial scleral thickness was measured starting at 3500–3600 μm posterior to the chamber angle, while the posterior scleral thickness was measured starting at 1000–1100 μm from the optic nerve. Example images indicating how the equatorial sclera and the posterior sclera thickness were shown in Fig 2B and 2C, respectively. The scleral thickness of each sample was measured at an interval of 200 μm using the ruler tool in in Leica Application Suite software, and 5 measurements were averaged.

HE stained sections were then observed and photographed with a light microscope (Leica DM-4000, Leica, Germany). The area observed by light microscopy was located 5000 μm posterior to the temporal chamber angle and the range was 500 μm. The thickness of the sclera, the choroid, and the retina were measured at an interval of 100 μm using the ruler tool in Leica Application Suite software, and 5 measurements from each sample were averaged.

### Expression of MMP-2 and MT1-MMP mRNAs

The right eyes were enucleated and cut circularly to remove the anterior segments, vitreous body, retina, and choroid. The temporal half of the sclera was harvested and then stored in liquid nitrogen. Total RNA was extracted from the temporal half of the sclera with Trizol reagent following the manufacturer’s instructions. All RNA samples were treated with DNase I (DNA-free Ambion, Darmstadt, Germany), and the respective yield was measured by spectrophotometry at 260 and 280 nm. The optical density (OD260/OD280) ratios were calculated to ensure the quality of the isolated RNA, and samples with a ratio between 1.8 and 2.0 were used for further analysis. This was followed by reverse-transcription of 1 μg of total RNA to 1st strand cDNA using the GoScript^™^ Reverse Transcription System (Promega Biotech Co., Ltd., USA) and the random primer provided in the kit, following the manufacturer’s instructions. The resultant cDNA was used as a template for qPCR, which was performed on a Roche LightCycler instrument using the LightCycler FastStart DNA Master SYBR Green I kit. The following protocol was used for the qPCR: initial denaturation at 95°C for 4 min followed by 40 cycles of denaturation at 95°C for 10 s, annealing at 60°C for 10 s and extension at 72°C for 20 s. The melting temperature curves of the PCR products were obtained. The mRNA expression of MMP-2 and MT1-MMP was normalized to that of an internal control gene, glyceraldehyde-3-phosphate dehydrogenase (GAPDH). We designed all of the PCR primers using Primer 3 software. The MMP-2 PCR product was 165 bp in length and the primer sequences were as follows: forward primer, 5’-GCT CTC TGC TTC CTG AGC TG-3’; reverse primer, 5’-AAC TTG ATG ATG GGC GAT G-3’. The MT1-MMP PCR product was 261 bp in length, and the primer sequences were as follows: forward primer, 5’-CCC AGA GCA ACT TCA G-3’; reverse primer, 5’-CCA GGA GGC AGG TAA CCA TA-3’. The GAPDH PCR product was 131 bp in length, and the primer sequences were as follows: forward primer, 5’-TCG CTC CTG GAA GAT GGT G-3’; reverse primer, 5’-TCAT TGA CCT CCA GTA CAT GG-3’.

### Western blot analysis of MMP-2 and MT1-MMP

Six guinea pigs in each group were selected for western blot analysis. Scleral samples were collected as described above for mRNA expression analysis and then homogenized with cold RIPA lysis buffer (Cowin Biotech Co., Ltd., Beijing, China). The lysate was centrifuged at 4°C (14000 rpm, 10 min) and the protein concentrations were determined using Bicin-choninic Acid (BCA) Protein Assay Kit (Cowin Biotech Co., Ltd., Beijing, China). Polyacrylamide gel was prepared for 10% sodium dodecyl sulfate polyacrylamide gel electrophoresis (SDS-PAGE). The protein was separated by SDS-PAGE and transferred onto polyvinylidene fluoride membranes. The membranes were blocked in 5% milk powder in Tris-buffered saline with Tween 20 (TBST) at room temperature for 60 min and incubated with primary antibodies overnight at 4°C and secondary antibodies at room temperature for 60 min. The primary antibodies were MMP-2 (orb592823, rabbit anti-guinea pig MMP-2 antibody, Biorbyt, UK) and MT1-MMP (LS-B11189-BCD1-50, goat anti-guinea pig MTI-MMP antibody, LSBio, USA). After the block and each incubation, the membranes were washed three times in TBST. The blots on the membrane were enhanced using enhanced chemiluminescence (ECL) and exposed to MiniChemi 610 Chemiluminescence Imaging System (Beijing Sage Creation Science Co., Ltd., Beijing, China) for scanning. The band density was analyzed using ImageJ software, and the relative expression normalized to GAPDH was calculated. The experiments were repeated three times with similar results.

### Statistical analysis

Statistical analysis was performed using SPSS software, version 17.0 (IBM-SPSS, Chicago, Illinois, USA). The data were presented as mean±standard deviation (SD). One-way ANOVA and Tukey’s multiple comparisons test were used to evaluate the differences among the three groups of eyes. In all tests, statistical significance was defined as p < 0.05.

## Results

### Ocular axial length and refraction

**Fig 3:**
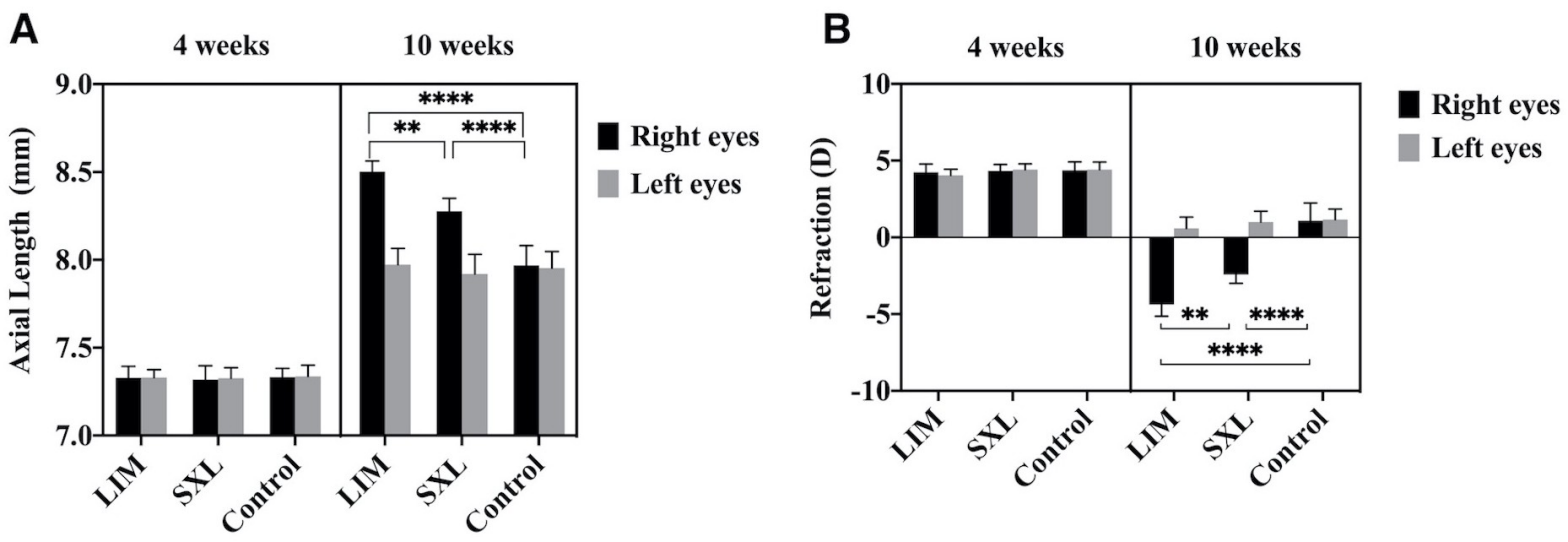
Ocular axial length and refraction assessment. (A) Significant differences in the mean AXL were observed among the three groups at 10 weeks of age (**p < 0.01 SXL vs. LIM, **** p < 0.0001 SXL vs. Control, ****p < 0.0001 LIM vs. Control). (B) The mean refraction was significantly different among the three groups at 10 weeks of age (**p < 0.01 SXL vs. LIM, **** p < 0.0001 SXL vs. Control, ****p < 0.0001 LIM vs. Control).

Fig 3 shows the AXL (Fig 3A) and refraction (Fig 3B) of the guinea pigs at baseline (4 weeks of age) and six weeks after the experimental treatments (10 weeks of age). At baseline, we found no differences in AXL between the left and right eyes in each any of the groups and no differences among the three groups of guinea pigs. After six weeks of lens wearing, myopia was successfully induced in the LIM group (8.50±0.06 mm, -4.38±0.77 D), as the guinea pigs in the LIM group had a significantly longer AXL and greater myopic refraction than those in the control group (7.97±0.12 mm, +1.08±1.15 D, p < 0.0001). The AXL and myopic refraction were significantly reduced in the SXL group compared with the LIM group (8.28±0.07 mm, -2.42±0.58 D, p < 0.01). The AXL and refraction of the left eyes were not significantly different among the groups at 10 weeks of age (p > 0.05).

### Light microscopy and regional scleral thickness

The data of equatorial and posterior scleral thickness are shown in Table 1. The sclera was thinner in all regions in the LIM group than in the control group. The temporal equatorial sclera and the temporal posterior sclera were significantly thicker in the SXL group than in the LIM group. The temporal equatorial sclera in the cross-linked groups was even thicker than that in the control group (p < 0.01). No significant differences in the nasal equatorial sclera and nasal posterior sclera thickness were found between the SXL and LIM groups (p > 0.05).

**Table 1:** Quantitative analysis data of light microscopy observations of the three groups of guinea pig eyes (mean ± SD, n = 5).

	LIM group	SXL group	Control group	P Values among 3 groups	P Values of Tukey’ s multiple comparisons test		
					Control vs. LIM	Control vs. SXL	SXL vs. LIM
Scleral thickness							
Temporal equatorial	32.17±5.09	60.15±13.20	48.66±7.27	< 0.0001	< 0.0001	< 0.01	< 0.0001
Temporal posterior	60.12±7.76	71.35±10.96	73.75±5.86	< 0.0001	< 0.0001	> 0.05	< 0.01
Nasal equatorial	36.14±7.80	38.47±4.73	44.63±7.71	< 0.05	< 0.05	< 0.05	> 0.05
Nasal posterior	64.08±6.89	69.40±4.14	79.78±7.85	< 0.0001	< 0.0001	< 0.05	> 0.05
Scleral thickness ^†^	48.58±5.28	67.30±5.49	65.35±4.73	< 0.0001	< 0.001	> 0.05	< 0.001
Choroidal thickness ^†^	18.19±2.10	24.39±3.44	25.42±2.53	< 0.001	< 0.01	> 0.05	< 0.05
Retinal thickness ^†^	75.58±4.99	74.29±2.80	77.37±6.73	> 0.05			

^†^ The observations and measurements area were located at 5000 μm posterior to the temporal chamber angle.

One-way ANOVA, Tukey’s multiple comparisons test.

**Fig 4:**
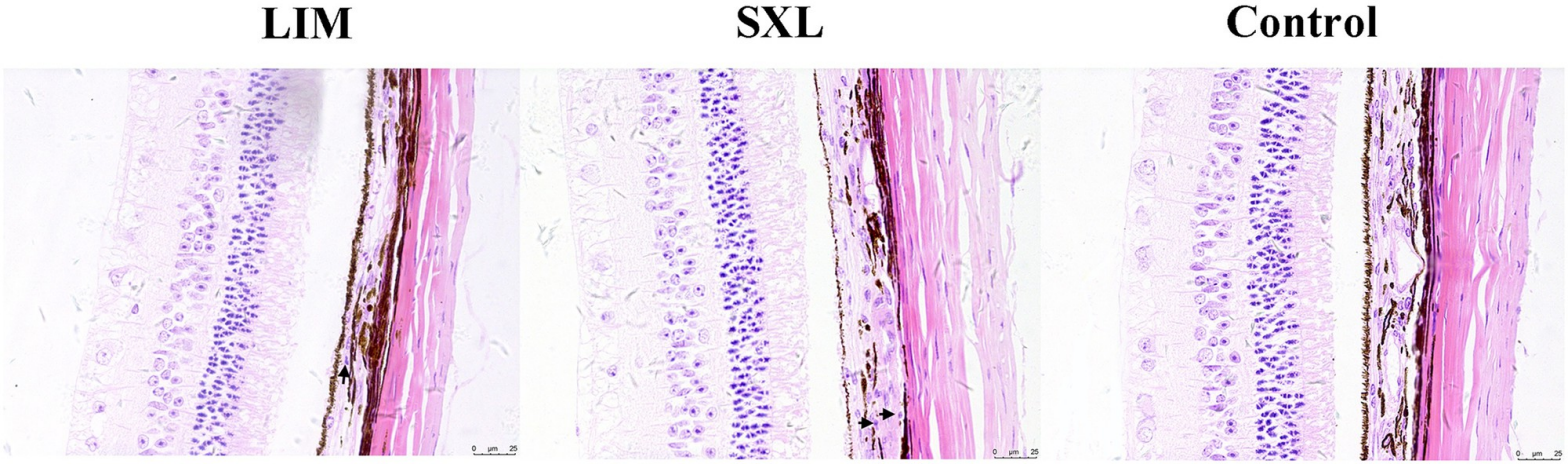
Magnified HE-stained photomicrographs of ocular tissue at 5000 μm posterior to the temporal chamber angle in the three groups. No obvious structural damage was observed in the sclera, choroid, or retina. The arrangement of scleral collagen fibers in the SXL group was similar to that in the control group. The collagenous tissue of the sclera was thinner in the LIM group than in the SXL and the control groups. The black arrows indicate a small number of chronic inflammatory cells scattered in the choroid. Scale bar: 25 μm.

The thickness of the sclera, choroid, and retina at 5000 μm posterior to the temporal chamber angle is presented in Table 1, and the magnified HE-stained photomicrographs of this area are shown in Fig 4. The scleral thickness of the LIM group was significantly lower than that of the SXL and control groups (p < 0.001), while no significant difference was observed between the SXL group and the control group (p > 0.05). The arrangement of the scleral collagen fibers was not obviously disorganized in the SXL group compared with the control group, and no obvious tissue edema or inflammatory cells were found in the cross-linked area. The choroidal thickness of the LIM group was significantly thinner than that of the control and SXL groups (LIM vs. control, p < 0.01; LIM vs. SXL, p < 0.05). In all samples from the three groups, a few chronic inflammatory cells, possibly lymphocytes and plasma cells (black arrows), were scattered within the choroid. The number of chronic inflammatory cells was not significantly different among the three groups (control group: 5.00±1.58, LIM group: 5.20±1.30, SXL group: 4.80±1.92; p > 0.05). There was no significant difference in retinal thickness among the three groups (p > 0.05). The cells in all retinal layers were arranged regularly, and we did not observe any morphological alterations in any of the three groups.

### Expression of MMP-2 and MT1-MMP mRNAs

**Fig 5:**
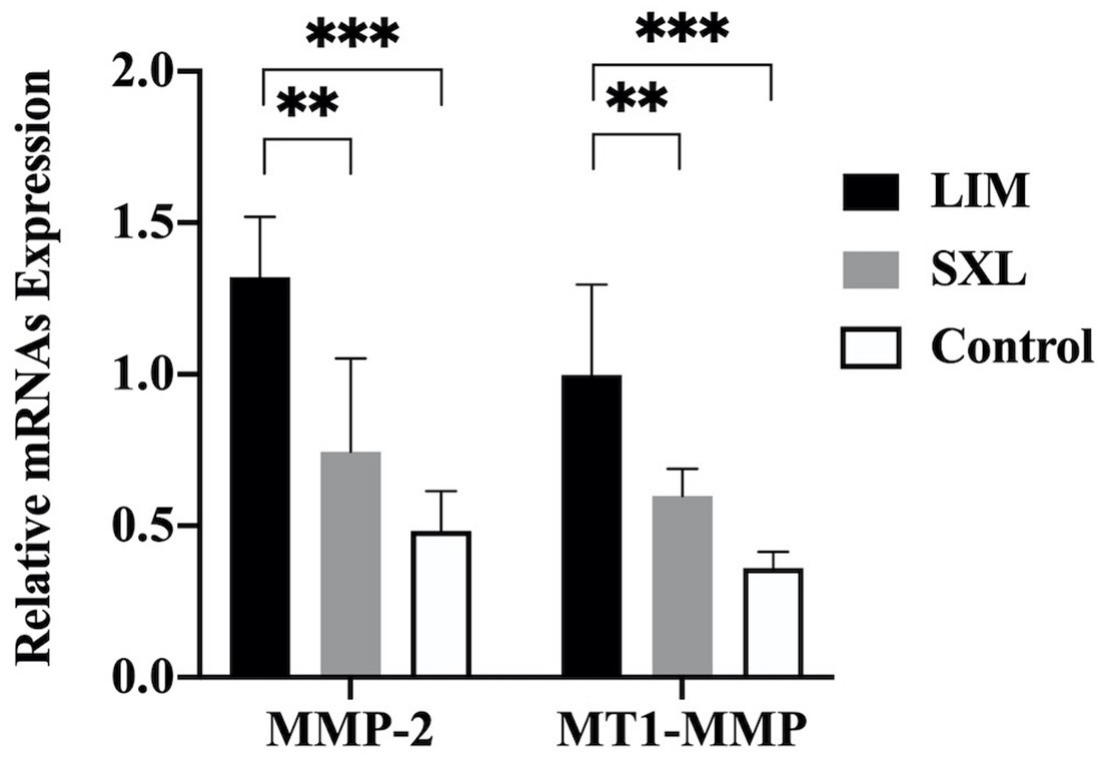
Relative expression of MMP-2 and MT1-MMP mRNAs in the temporal sclera of the three groups of eyes. Significant differences in the expression of MMP-2 and MT1-MMP were observed between the LIM group and the other two groups (**p < 0.01 LIM vs. SXL, ***p < 0.001 LIM vs. Control, p > 0.5 SXL vs. Control).

The relative expression of MMP-2 and MT1-MMP mRNAs in the sclera on the temporal side is shown in Fig 5. MMP-2 and MT1-MMP mRNA levels were significantly higher in the LIM group than in the control group (both p < 0.001). We found that the mRNA expression of MMP-2 and MT1-MMP was significantly lower in the SXL group than in the LIM group (both p < 0.01). The difference in mRNA levels between the SXL and control groups was not significant.

### Expression of MMP-2 and MT1-MMP protein

**Fig 6:**
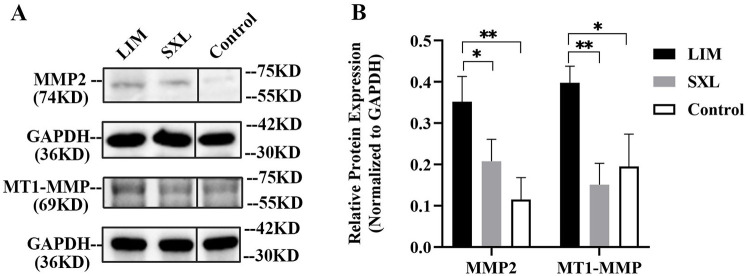
Expression of MMP-2 and MT1-MMP protein in the temporal sclera in the three groups of eyes. (A) Representative western blot image. The lanes of samples from the three groups were rearranged to plot a figure showing expression in the three groups. The vertical black lines denote the splicing position where the bands were cut from the same blot. (B) The differences in the protein expression of MMP-2 and MT1-MMP between the LIM group and the other two groups were significant (MMP-2: *p < 0.05 LIM vs. SXL, **p < 0.01 LIM vs. Control, p > 0.05 SXL vs. Control; MT1-MMP: **p < 0.01 LIM vs. SXL, *p < 0.05 LIM vs. Control, p > 0.05 SXL vs. Control).

The expression of MMP-2 and MT1-MMP protein in the sclera on the temporal side is shown in Fig 6. MMP-2 and MT1-MMP protein levels were significantly higher in the LIM group than in the SXL and control groups (p < 0.05). There was no significant difference in MMP-2 or MT1-MMP protein expression between the SXL and control groups (p > 0.05).

## Discussion

In both human myopia and animal models of experimental myopia, the scleral thickness has been found to become thinner with excessive axial growth [23–25]. In this study, excessive axial elongation, scleral thinning, and increased MMP-2 and MT1-MMP expression were found in the LIM group, which may suggest that scleral remodeling occurred in lens-induced myopic guinea pigs.

In our study, the manipulation of riboflavin/UVA SXL before myopia induction could prevent the myopia progression and increase the scleral thickness. Our result was comparable to the finding of Choi et al. [26] that the riboflavin/UVA collagen cross-linking could increase the thickness of the human cornea and sclera. In the SXL group, the temporal equatorial sclera exhibited thickening to a greater extent than the temporal posterior sclera. This difference might be related to the thinner scleral thickness at the equatorial sclera than at the posterior sclera. The greater infiltration degree of cross-linking with riboflavin/UVA may result in the more significant effects of cross-linking on scleral thickening at the equator. It has been demonstrated that the thickness change of the posterior sclera is likely to be related to myopia-induced scleral remodeling in marmosets and tree shrews [23, 24, 27]. However, few studies investigated the effects of the equatorial sclera. Girard et al. [28] hypothesized that the biomechanical properties of equatorial meridional scleral fibers might limit the axial elongation in rats. It has been reported that the biomechanical properties of sclera was reinforced by SXL [19]. Thus, we speculate that collagen cross-linking at the equatorial sclera may also inhibit AXL elongation and further exploration is needed. We found that the scleral thickness was greater in the SXL group than in the control group at the equator sclera. The previous studies have reported that after riboflavin/UVA SXL intervention, the scleral fibroblasts were rich in the rough endoplasmic reticulum, and the expression of collagen I gene was increased in rabbits [29], and ECM degradation by MMPs were inhibited in bovine cornea [30]. We hypothesize that scleral thickening occurs because SXL might improve the synthesis of collagen and suppress ECM degradation by MMPs.

The areas observed and measured by light microscopy were located between the equatorial and the posterior sclera. The scleral thickness in this area is consistent with the posterior scleral thickness. Choroid thinning was observed in the LIM group, which is consistent with previous studies in guinea pig models of lens-induced myopia [31, 32]. Moreover, Lu et al. [32] found that the recovery of myopia in guinea pigs was accompanied by choroid thickening. The choroidal thickening in the SXL group might therefore be partly associated with the inhibition of myopia progression in this study. We found that there was no significant difference in the retinal thickness among the 3 groups. This is in agreement previous results that the development of experimental myopia does not affect retinal thickness in guinea pigs [31, 32]. These results are also consistent with studies in rhesus monkeys, in which the retinal thickness showed no significant difference after the riboflavin/UVA SXL [33]. Some of our previous works have investigated the safety of riboflavin/UVA SXL (365 nm, 3 mW/cm2, 30 min) in rabbits and rhesus monkeys. The electroretinography amplitudes were significantly reduced and the photoreceptor cells were damaged in rabbits [34]. However, the electroretinography amplitudes were not affected and no histopathological abnormalities were found in rhesus monkeys 1 year after SXL [33, 35, 36]. In this study, light microscopy observations indicated that riboflavin/UVA SXL did not result in obvious structural changes in the sclera, choroid, or retina. Despite this, our histological results have limitation. The inflammatory cells were identified by HE-staining, study of study on inflammatory cell markers may verify the inflammation further.

MMP-2, as the most studied member of the MMP family in the sclera, is capable of degrading a range of components of scleral ECM, including collagens and proteoglycans [37, 38]. Increases in the expression and activity of MMP-2 are associated with increased scleral collagen degradation and scleral thinning in both human and animal myopia [3, 4]. This activation of proMMP-2 is dependent on the relative levels of MT1-MMP. Siegwart and Norton [39] found that the MT1-MMP and MMP-2 expression was up-regulated in lens-induced myopic eyes relative to control eyes in the tree shrews. The expression of MT1-MMP and MMP-2 was also up-regulated in guinea pigs in the LIM group in the present study, which may suggest that this mechanism is also active in the guinea pig lens-induced myopia models. MT1-MMP is also a multi-function proteinase that can cleave various ECM macromolecules that take part in the ECM metabolism [40, 41]. Fini et al. [42] demonstrated that the levels of MMPs are significantly altered at the level of transcription. In our study, the mRNA and protein of both MT1-MMP and MMP-2 were increased in guinea pigs in the LIM group, suggesting that scleral remodeling might be active afterward, contributing to the thinning of the sclera.

Seseogullari-Dirihan et al. [43] and Cova et al. [44] indicated that riboflavin/UVA collagen cross-linking inactivated dentin MMPs and reduced the degradation of dentin collagen. It is also shown that the protein level of MMP-2 was decreased by whole-body UVA irradiation plus oral administration of riboflavin in the guinea pig model of lens-induced myopia [45]. It is consistent with our result that the mRNA and protein of MMP-2 and MT1-MMP were lower in the SXL group than in the LIM group. We speculate that riboflavin/UVA SXL could downregulate the expression of MMP-2 and MT1-MMP to reduce ECM degradation. The decrease of MT1-MMP expression may also inhibit the action of proMMP-2 and eventually weaken its ability to degrade ECM. The thickening of the cross-linked sclera may partly be caused by the reduction of ECM degradation. Altered expression of MMP-2 and MT1-MMP may be one of the mechanisms underlying the protective effects of SXL in the guinea pig myopia model.

In conclusion, the present findings suggest that riboflavin/UVA SXL could slow AXL elongation and decrease the degree of myopic refraction. The thickening of the cross-linked sclera might be partly related to the downregulated expression and decreased activity of MMP-2 during the scleral remodeling process in lens-induced myopic guinea pigs.
